# Prevalence of cervical and anal warts among HIV patients on ARV Nigerian special treatment center

**DOI:** 10.1186/1750-9378-7-S1-O13

**Published:** 2012-04-19

**Authors:** Modupe Onigbogi, Omobola Ojo, Olanrewaju Onigbogi, Oluwaseun Akinyemi

**Affiliations:** 1 Department of Community Health and Primary Care, CMUL, University of Lagos, Nigeria; 2 Department of Community Medicine, University College Hospital, Ibadan, Nigeria

## Background

With the introduction of HAART in many HIV treatment centers in Nigeria, there has been a decreased incidence of AIDS-related mortality and growing concern about the incidence of AIDS specific and non-AIDS specific cancers. This study thus sought to investigate patterns and incidence of cervical and anal warts among patients in a Special Treatment Center (SPC) before being diagnosed of having HIV and after starting ARVs.

## Material and methods

We linked results from a population-based HIV registry in our SPC with that of two cancer registries and evaluated the risk of developing anal or cervical warts a year prior to onset of ARV and a year after commencement of HAART. Standardized incidence ratios (SIRs) were calculated to relate presence of cervical and anal warts in people with AIDS to that in the general population. We also used logistic regression with 95% confidential interval to compare risk according to demographic factors and CD4 count.

## Results

The study involved 542 patients diagnosed with HIV within the two years of the study. Persons with AIDS had elevated risks of cervical warts (SIR=11.7, 95% CI 6.2-14.4, n=52) and anal warts (SIR=1.8, 95% CI 1.3-2.7, n=13). Risk for cervical warts increased with increasing time relative to AIDS onset (p=0.02), this trend was not significant for anal warts (p=0.12). Risk of developing both warts was unrelated to CD4 count at onset of treatment (p=0.43).

## Conclusions

There is a modest excess risk of developing cervical and anal warts among this group of patients. The risk seems to increase with increasing years on ARVs. This is not related to the immune state of the patients. There is a need to pay attention to patients on ARV therapy as they advance in years with ARV medication.

